# Blastemal predominant WT1 negative Wilms tumour of the young adult: a unique case report and review of the literature

**DOI:** 10.3389/fmed.2025.1507011

**Published:** 2025-03-19

**Authors:** Jozef Škarda, Michal Grepl, Valeria Skopelidou, Vladimír Židlík, Pavel Hurník, Daniela Skanderová, Michael Michal, Michal Michal, Pavla Hanzlíková, Jana Vaculová, Marcel Mitták

**Affiliations:** ^1^Institute of Molecular and Clinical Pathology and Medical Genetics, University Hospital Ostrava, Ostrava, Czechia; ^2^Institute of Molecular and Clinical Pathology and Medical Genetics, Faculty of Medicine, University of Ostrava, Ostrava, Czechia; ^3^Department of Urology, University Hospital Ostrava, Ostrava, Czechia; ^4^Department of Surgical Studies, Faculty of Medicine, Ostrava University, Ostrava, Czechia; ^5^Department of Pathology, EUC Laboratoře CGB a.s., Ostrava, Czechia; ^6^Department of Pathology, Faculty Hospital Olomouc, Olomouc, Czechia; ^7^Department of Pathology, Faculty of Medicine and Dentistry, Palacky University Olomouc, Olomouc, Czechia; ^8^Department of Pathology, Faculty of Medicine, Charles University, Plzen, Czechia; ^9^Bioptical Laboratory Ltd., Pilsen, Czechia; ^10^Department of Radiology, University Hospital Ostrava, Ostrava, Czechia; ^11^Department of Imaging Methods, Faculty of Medicine, University of Ostrava, Ostrava, Czechia; ^12^Department of Surgery, University Hospital Ostrava, Ostrava, Czechia

**Keywords:** renal, Wilms tumour, nephroblastoma, adult, WT1 negative, blastemal predominant, case report

## Abstract

Wilms tumour is a common juvenile cancer of the kidney, and its occurrence in adolescence or adulthood is extremely rare, accounting for around 1% of all adult kidney malignancies. Histopathologically, three tissue patterns can be identified, including blastemal, epithelial, and stromal components, while the overall microscopic appearance of an adult-type tumour does not differ from that of its juvenile counterpart. The blastemal predominant Wilms tumours are the most aggressive and have the worst prognosis. The samples must be histopathologically verified before the definitive diagnosis can be made, and immunohistochemistry examination is critical. Wilms tumours are often positive for keratin, vimentin, desmin, actin, and WT1, which distinguishes this type of tumour from other malignancies. WT1 positivity is indicative of the blastemal component of the tumorous tissue and may be completely absent in the mature epithelial and stromal parts. Only three WT1 negative adult-type Wilms tumours have been reported in the literature to this date. However, none of the patients had a blastemal predominant tumour. That is why we would like to present a highly interesting and diagnostically challenging case of a young man who was diagnosed with a tumorous lesion of the left kidney parenchyma. Genetic analysis did not reveal any known fusion genes associated with round cell sarcomas, ruling out this differential diagnosis. This article also includes a literature review on published articles on WT1 negative Wilms tumour in adults and other concerns related to this topic. The main goal of this publication was to emphasise that, while it is a rare entity in general, similar problematic cases can occur in practise, and thus it is important to be aware of this type of tumour when making a differential diagnosis in cases with similar clinical and histopathological features.

## Introduction

Wilms tumour (nephroblastoma) is a typical renal tumour of childhood. The median age of diagnosis is 3–4 years, and 90% of cases are diagnosed before the age of 7. It is considered to be one of the embryonic tumours due to its microscopic morphology. Wilms tumour in adolescence or adulthood is extremely rare, accounting for about 1% of all kidney malignancies in older age. Adult nephroblastoma is currently understudied due to its rarity, as seen by the low number of quality studies and documented case reports (only about 300) (1–4).

Histopathologically, three tissue patterns can be found, including blastemal, epithelial, and stromal components. The overall microscopic appearance of an adult-type tumour does not differ from its juvenile counterpart. Tumour tissue composition is one of the key variables for predicting risk and may help to determine the prognosis of patients with WT. In favourable tumours, all three components and focal or absent anaplasia are found in the tumour; on the other hand, tumours with a blastemal component predominance or the presence of diffuse anaplastic changes are considered high risk (according to SIOP 2001 – Societe Internationale D’oncologie Pediatrique). The so-called blastemal predominant Wilms tumours are the most aggressive and have the worst prognosis (5–8).

Kilton et al. developed pathological criteria for adult-type nephroblastoma that included age over 15 years, lesions primarily arising from renal tissue, histological features of embryogenic glomerulotubular structures with immature spindle or round cell stroma, and the absence of tissue corresponding to renal cell carcinoma. The ensuing diagnosis should be validated histopathologically, and immunohistochemical investigation is also crucial. Nephroblastomas are generally positive for keratin, vimentin, desmin, actin, and WT1, allowing this form of tumour to be distinguished from other unusual entities. However, WT1 positivity is typical especially for the blastemal component of the tumorous tissue and may be completely absent in the mature epithelial and stromal parts (6, 9–11).

To date, only three WT1 negative adult-type Wilms tumours have been documented in the literature (Table 1; one of them reported non-specific expression). However, not a single patient had a blastemal predominant tumour.

**Table 1:** Review of all reported WT1 negative adult renal Wilms tumour cases in the current literature.

Case	Year	Country	Age/Sex	Initial presentation	Localization	Maximum size (cm)	Predominance	Immunohistochemical staining	SIOP risk	Reference
1	2025	Czechia	21/M	abdominal pain, acute internal bleeding from the tumorous tissue	lower segment of left kidney	8,5	Blastemal	KRT7+, CD10+, PAX8+, PAX2+, OSCAR+, KRT18+, TTF1+, INI1+, KRTAE1/AE3+, p63+, GPC3+ Syn−, Vimentin−, KRT5/6−, CD34−, CD56−, Chromogranin−, AMACR−, RCC−, **WT1−**, LCA−, CD20−, desmin−, STAT6−, Inhibin alfa−, CD30−, CD99−, Calcitonin−, S100−, SOX10−, melan A−, PHOX2B−, SS18−, SSX−, PSA−, NKX3-1−	Intermediate	Current case
2	2022	Austria	40/M	persistent high blood pressure	right kidney	25,5	Epithelial	KRTAE1/AE3+**WT1−**	Intermediate	(1)
3	2022	China	25/M	incidental finding (admitted for a planned knee operation)	lower segment of left kidney	5,2	Epithelial (?)	Ckp+, CD34+, CD56+, PAX8+, S100+ Vimentin−, **WT1−**, Syn−, CgA−, RCC−, TFE3−, NeuN−, GFAP−, CD10−, EMA−, Inhibin alfa−	High	(31)
4	2020	Iran	39/F	persistent pain in the right flank	upper half of right kidney	4	Epithelial	EMA+, **WT1 non-specific** KRT−, CD56−, CD10−, CD99−	Intermediate	(2)

The given table summarizes existing cases of patients with adult-type renal Wilms tumour with proven WT1 negativity. Abbreviations used (excluding immunohistochemical staining): F – female, M – male, SIOP – Societe Internationale D’oncologie Pediatrique (International Paediatric Oncology Congress). Bold text highlights WT1 expression in given cases.

We present a diagnostically challenging case of a young adult man in whom a tumorous lesion of the parenchyma of the left kidney presented as an acute bleeding. After a complex diagnostic process, it was discovered to be an adult WT1-negative Wilms tumour with blastemal predominance. This case report includes a detailed immunohistochemical and molecular-genetic analysis, contributing to the classification of this rare nosological unit. The case report itself was written according to the CARE checklist (Supplementary material 1).

## Case description

We present the case of a 21-year-old man, who was admitted to the general surgical outpatient clinic of Ostrava University Hospital for sudden severe abdominal pain in the left hypogastrium. The discomfort remained consistent (with no respite when shifting positions) and did not radiate. The patient consumed around 2 litres of fluids in the morning but was unable to urinate for several hours before the appointment, denying other urological symptoms. Otherwise, he was a healthy individual with no similar prior issues.

The patient was stable, afebrile, slightly nauseated, fully oriented, and cooperative upon admission. The abdomen was soft and palpable on physical examination, however probing on the left side (left hypogastrium and area above the pubic clasp) caused intense pain. Furthermore, the Plénies’ sign was determined to be positive, percussion on the left was borderline positive (negative on the right). Other findings were not clinically significant.

**Figure 1:**
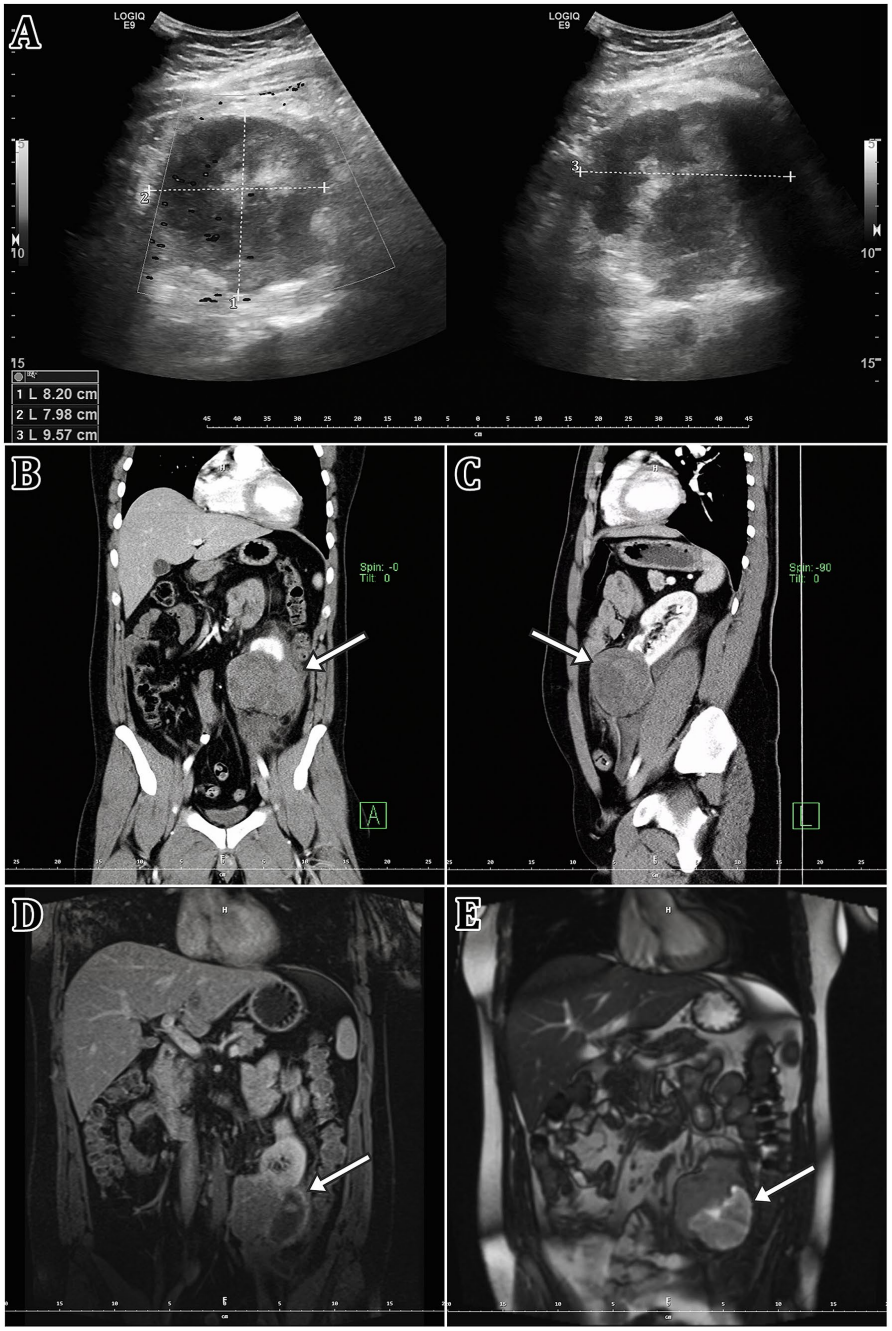
This figure summarises the findings of the primary tumorous focus’s imaging modalities. **(A)** Ultrasound imaging, **(B,C)** Computed tomography imaging (CT; contrast enhanced), **(D,E)** magnetic resonance imaging [MRI; **(D)** T1, **(E)** T2 sequence]. A white arrow indicates the tumorous lesion itself.

The patient was immediately taken for an ultrasound, which revealed a hypoechoic focus near the lower pole of the left kidney (Figure 1). However, the quality of the ultrasound examination was limited, thus it was decided to perform a contrast CT examination of the abdomen and pelvis area (Figure 1). There was a well-defined pathological expansion in the lower pole of the left kidney measuring 70 × 88 × 75 mm. Numerous fluid collections were also observed around the left kidney. Other pathological alterations, such as lymph node enlargement or indications of inflammation or active bleeding, were not observed.

The patient was then admitted to the Urology Clinic’s standard inpatient department, where he was treated conservatively. The pain lessened and his overall clinical condition improved during the hospitalisation, and he was discharged home on the 7th day. Following the radiologist’s recommendation, a contrast MRI follow-up evaluation of the kidney findings was carried out three weeks after discharge from the hospital (Figure 1). The dominant finding was a T2 mixed-signal expansion of the caudal part of the left kidney up to 74 mm in size. The lesion contained numerous cystic structures and breakdown products of blood were evident. The solid component showed T2 hyposignality. Other findings were normal.

Due to extremely concerning findings, a biopsy of the left kidney was performed under CT guidance, and the samples were sent for rapid histopathological evaluation to the Departments of Clinical and Molecular Pathology and Medical Genetics. The acquired tissue microfragments exhibited morphological characteristics of a malignant tumour, including a population of small round cells with a small amount of cytoplasm. Immunohistochemical analysis revealed KRTAE1/3 (dot-like) positivity, weak GPC3 and PAX8 positivity, and up to 70% Ki67 proliferative activity. CD3, CD20, LCA, EMA, WT1, synaptophysin, chromogranin, and CD56 markers were all negative. Further specification of the findings was not possible for the small amount of the sample.

**Figure 2:**
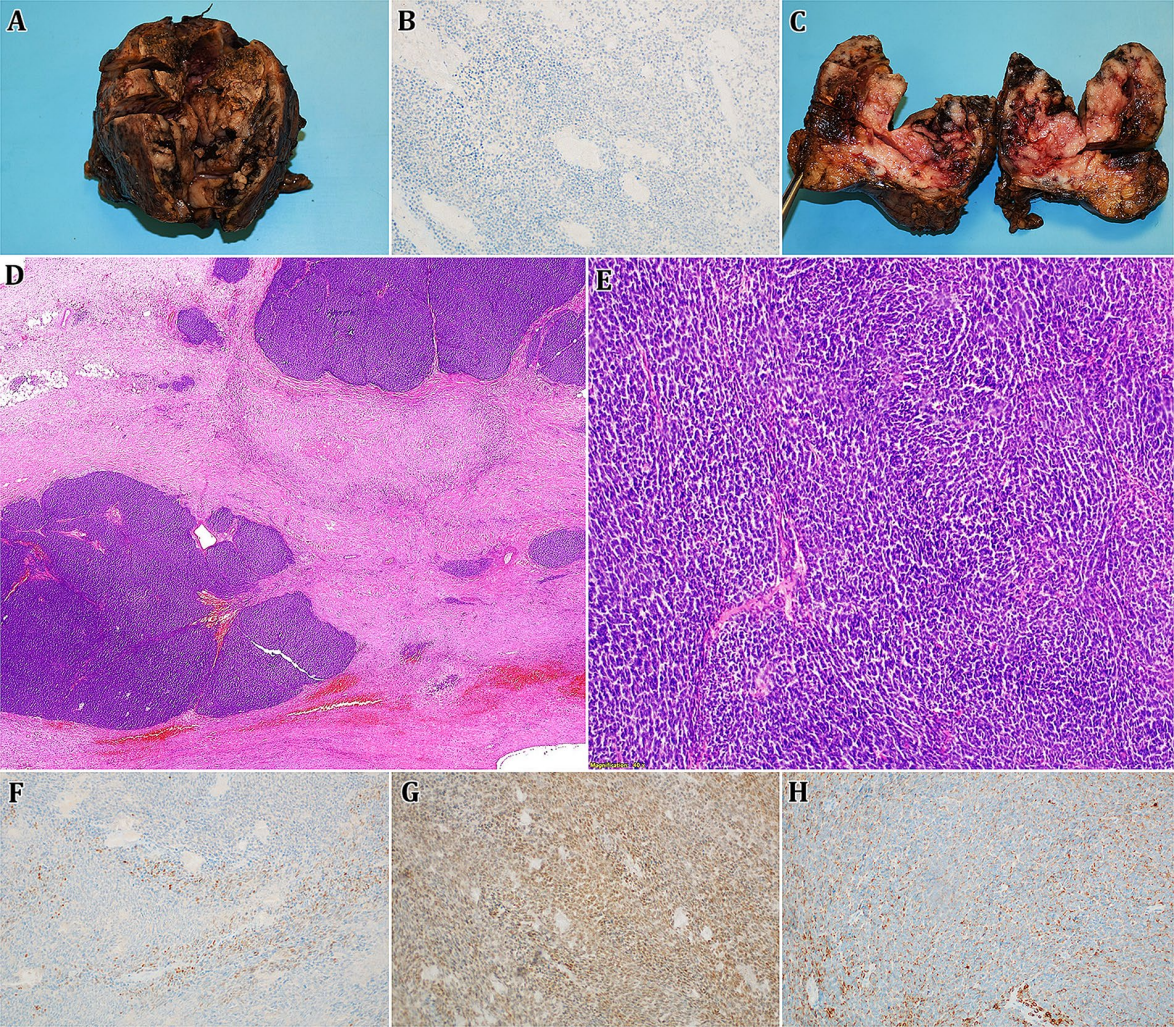
Given figure consists of an images **A**,**C** representing a macroscopic finding of a surgically resected lesion (bulky tumorous mass of an inhomogeneous structure with haemorrhage, discoloration, and focal friability), **B,D-H** then show microscopic structure of obtained slides (**D,E** – haematoxylin-eosin staining, 0,5x and 100x magnification; **B,F-H** – immunohistochemistry methods, 100x magnification). Microscopically, cross-sections of tumorous tissue show spindles of a weakly differentiated, partially necrotizing tumour with high mitotic activity and notable nuclear polymorphism. Immunohistochemistry confirmed the positivity of p63 (weak; **F**), PAX8 **(G)**, and KRT7 (limited focal; **H**), whereas WT1 was negative **(B)**.

Following that, surgical resection of the left kidney tumour was performed. The excised tumour was subsequently sent for further pathological investigation. Macroscopically, a specimen weighing 250 g and measuring 8.5 × 7 × 6.5 cm was observed, where a bulky tumorous lesion of an inhomogeneous structure with a total size of 7 × 5.5 × 5 cm was found on the section. The lesion showed extensive haemorrhage, discoloration, and focal friability. The key microscopic finding was the spindles of a poorly differentiated, partially necrotizing tumour with high mitotic activity and significant nuclear polymorphism. Immunohistochemically, the CD10 positivity and limited focal positivity of KRT7 was confirmed, on the other hand, the expression of synaptophysin, vimentin, KRT5/6, CD34, CD56, chromogranin, AMACR, CD99, RCC and WT1 (N-terminal specific antibody) were negative (Figure 2). The proliferative Ki67 index did not exceed 15%, whereas in the initial biopsy, a 70% proliferative activity was observed, which may reflect intratumour heterogeneity and sampling differences.

**Figure 3:**
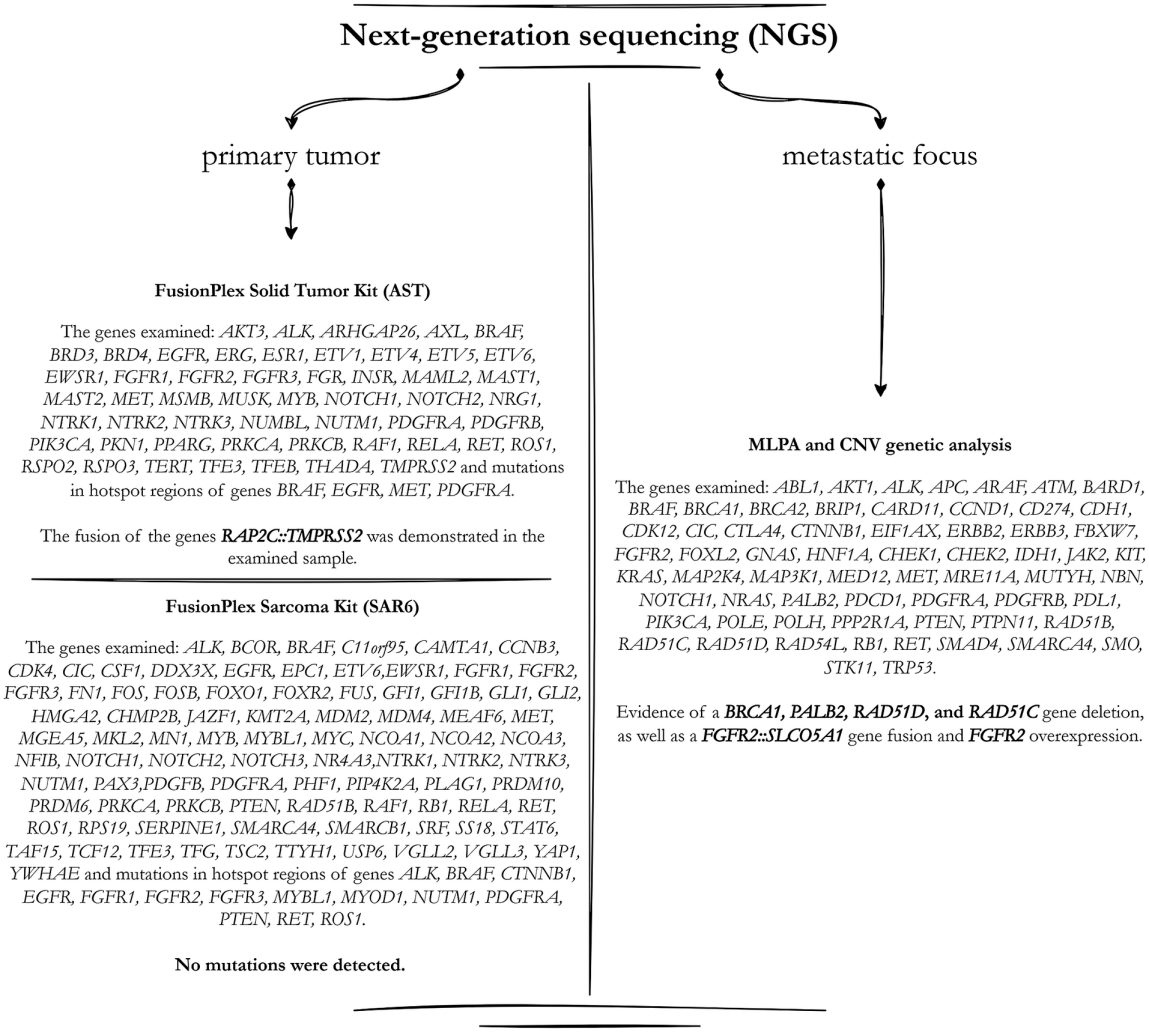
The scheme describes the detailed genetic analysis carried out utilising the NGS (next-generation sequencing) method. The left section summarises the analysed genes and mutations discovered during the primary tumour sample evaluation. The right part correlates to the results from the secondary metastatic focus.

Due to the ambiguity of the histopathological appearance, the samples were sent to the Department of Clinical and Molecular Pathology at Olomouc University Hospital for consultation. The result was classified as a poorly differentiated carcinoma, with a morphological appearance that suggests neuroendocrine differentiation, although no neuroendocrine markers were identified. PAX8 expression was found, indicating a primary origin in the kidney, however more examination was required due to TTF1 positivity, which, along with PAX8, could imply a metastatic origin of the lesion. It was decided to send the samples for the third reading to the Bioptic Laboratory Ltd. in Pilsen. An extensive molecular-genetic analysis (NGS) was undertaken with the goal of discovering most of the known diagnostic fusion genes seen in sarcomas – results were however negative (Figure 3), indicating that it was not a sarcoma (neither its round cell subtype). Based on the morphology and the results of the immunohistochemical examination, two differential diagnoses were considered – undifferentiated renal cell carcinoma and an adult form of Wilms tumour. Due to the localization, the patient’s young age, the morphology of the tumour tissue, and the immunoprofile (PAX8+, TTF1+, GPC3+, PAX2+, and RCC-), adult non-anaplastic Wilms tumour with blastemal predominance was the final diagnosis (despite WT1 negativity). There was no epithelial nor stromal component present. The discovery was then classified as intermediate risk by SIOP.

After the partial kidney resection, no evidence of local recurrence of the disease was identified during the three-month follow-up CT examination, but a newly formed, suspicious lesion in the parenchyma of the left adrenal gland was observed. A laparoscopic adrenalectomy was performed but no tumour was histologically proved (only extensive fibrosis with granulomatous reaction), indicating that the clinical finding was still pT2NxM0. The consequence of this procedure was the development of systemic hypertension, the patient was prescribed ACE-inhibitors with a satisfactory effect. The following step was a follow-up PET/CT scan (7 months after tumour removal), which revealed substantial cancer progression. In the zone of mild fibrous alterations, a suspicious nodulation was observed on the transverse sheet of Gerota’s fascia on the left. Furthermore, radiopharmaceutical activity was seen in the axillary and cervical lymph nodes, as well as the thymic parenchyma, however this was most likely due to reactivity following COVID19 immunisation. An MRI examination followed (14th month), during which additional metastatic foci were revealed – behind the lower pole of the left kidney ventrally and dorsally (in the fatty capsule), another one near the left iliac artery, and a focus on the psoas muscle, the size of which was 28 × 26 mm, was also confirmed. There was a tightening of the jejunal loop (without signs of tumorous infiltration).

The decision was made to remove the greatest metastatic focus on the left psoas through the left pararectal incision, which was done without any compilations. The specimen was sent for histopathological evaluation, where the microscopic image was identical to the image of the primary tumour. Immunohistochemical positivity for p63 (weak), KRT7 (limited focal), PAX8 (strong), KRT18 and CD99 (membranous) was observed. Tumour structures did not express WT1 or AMACR positivity. PD-L1 expression was also negative with combined positive score < 1. Due to morphological and histopathological findings it was thus confirmed that it was indeed a metastasis of a primary non-anaplastic Wilms tumour. An NGS examination was also performed, the results of which showed that no clearly pathogenic sequence variant was found in any of the examined genes (Figure 3). However, several fusion genes, particularly the F*GFR1::SLCO5A1* fusion, have been found. High *FGFR1* expression and *FGFR2* overexpression were also observed.

The patient was transferred to the outpatient care of the Paediatric Oncology Clinic of the Brno University Hospital, where systemic therapy was started. The patient underwent combined chemo-and radiotherapy according to the Umbrella SIOP-RTSG 2016 protocol, primarily designed for paediatric patients but also relevant for rare tumours in adults. Unfortunately, a more precise description of the therapeutic procedures is not available. This treatment started one year after the first surgical procedure, mainly because of the complicated and therefore rather time-consuming process of establishing the final histopathological diagnosis. Following which, the patient reached complete remission and continues to be followed without signs of cancer relapse.

## Discussion

Wilms tumour in adults is a rare entity of renal malignancies, which is also connected with a lack of information and studies on this topic. This is also related to the fact that it is still unclear if the adult and juvenile Wilms tumours represent the same biological unit and are equivalent. Less than 3% of all nephroblastomas are diagnosed in adulthood, making it a difficult entity to diagnose and treat, but also to research (1–3).

Clinically, adult Wilms tumour often manifests itself as flank or abdominal pain, palpable abdominal mass, haematuria, hypertension, or coagulopathy. Most commonly, however, it is an incidental finding when performing imaging methods for another reason. Targeted diagnosis of Wilms tumour is very complicated, since detecting a tumorous mass using imaging modalities does not distinguish this entity from other malignancies with similar morphology. Adult nephroblastoma is notably difficult to differentiate from renal cell carcinoma, which is a considerably more likely diagnosis (5, 10–12).

Histopathological evaluation of tumorous tissue samples is thus crucial. There is no morphological difference between the adult type of nephroblastoma and its juvenile counterpart (however, it is important to follow the methodology by Kilton et al.). Nephroblastomas are composed of blastemal, epithelial, and stromal components, but all three are rarely present at the same time. Furthermore, one of the components tends to dominate, which is referred to as component predominance. If the epithelial or stromal component predominates, the nephroblastoma is less aggressive, and the patient’s prognosis is better than in subtypes with blastemal predominance. The presence of poorly differentiated and highly malignant tissue comprised of small round cells with overlapping nuclei and intense mitotic activity characterises blastemal predominance. This subtype must be distinguished from other tumours with a similar morphology, particularly neuroendocrine tumours or hematogenous malignancies. This component’s lack of differentiation can also imitate Ewing sarcoma or neuroblastoma. It is also necessary to evaluate the occurrence of cellular anaplasia, which is distinguished by the presence of multipolar mitotic figures and enlarged hyperchromatic nuclei. It can be found in up to 10% of all nephroblastomas. It is also crucial to specify whether the anaplasia is focal or diffuse, as this has a significant impact on the patient’s prognosis, risk of relapse, and overall outcome (6, 8, 13, 14).

The use of immunohistochemistry methods is critical in making a definitive diagnosis. It mostly serves in distinguishing distinct units with similar morphology and microscopic appearance. The examined markers are CD56, CD57, WT1, AMACR, KRT7, and KRTAE1/AE3, which assist to separate nephroblastoma with epithelial predominance from morphologically indistinguishable metanephric adenoma. Keratins and WT1 are expressed in more than 50% of cases of MA, while AMACR is positive in only 10% of cases and CD56 is negative. The hallmark of metanephric adenoma is the presence of the *BRAF V600E* mutation, seen in up to 90% of cases, which makes it useful diagnostically when attempting to differentiate these two tumours. Nephroblastoma generally does not harbour mutations of *BRAF* but tends toward other genetic alterations, including, but not limited to *WT1* and *CTNNB1* mutations. Wilms tumours with blastemal predominance are positive for CD56, CD57, and WT1 in the great majority of patients, although the findings must be distinguished from lymphomas, sarcomas, or less likely metastases from other regions. It is desirable to assess the expression of chromogranin, synaptophysin, and other previously indicated markers in this context. Furthermore, the expression of vimentin, desmin, actin, and other molecules is examined; the resulting spectrum of potential units is extremely diverse and is dependent on which subunits must be excluded. Evaluation of the proliferative Ki67 index does not have an established typical value that could be authoritative in the diagnosis of nephroblastoma. Also, the results of the studies differ, some authors state that the blastemal component is the most proliferatively active, while others claim that the epithelial component is the most proliferative. For smaller tissue biopsy samples, assessing WT1, CD56, CD57, CD20, KRT18, KRT8 and EMA seem appropriate. WT1, which is positive in more than 90% of all nephroblastomas, especially those with a blastemal and proliferative epithelial component, is one of the most helpful markers (2, 5, 11, 13–18). However, as demonstrated by our case, this is not always true, and even cases with blastemal predominance can be WT1 negative, necessitating a more in-depth understanding of the issue.

A molecular-genetic examination is also important, as there is an immense amount of gene mutations connected with the risk of developing nephroblastoma, particularly in children. In most cases, this includes genes involved in kidney development, which only adds to the overall embryonic explanation of nephroblastoma formation – for example *WT1, WT2*, the *IGF2* region, the *WNT* pathway, *MYCN, WTX, CTNNB1*, and *TP53*. Recently discovered ones include miRNA processing genes and the transcription factors *SIX1/2* (3–5, 7, 19–23). In our case, an extensive NGS examination was performed, however, the clinical significance of our findings remains uncertain, as tumours with complex genetics can harbour structural rearrangements that do not necessarily drive tumorigenesis. Given that the *FGFR1::SLCO5A1* fusion was detected in the primary tumour but not in the metastatic focus, it is possible that this represents a random event rather than a pathogenic alteration. Additionally, observed deletions could indicate genomic instability rather than functionally relevant alterations. The absence of WT1 expression, lack of characteristic genetic alterations and the presence of atypical KRT7 positivity raise the question of whether this tumour represents a true nephroblastoma or a morphologically similar but biologically distinct neoplasm. KRT7 expression is typically restricted to epithelial components, with Kinney et al. (24) reporting positivity in only 1 of 20 epithelial-predominant cases, and Argani et al. (25) finding it absent in adult tumours. Its focal presence in our blastemal predominant tumour is therefore an unusual finding. Some studies suggest that adult Wilms tumours may encompass a spectrum of renal embryonal tumours with nephroblastoma-like differentiation rather than being direct counterparts of paediatric nephroblastoma. Further molecular and transcriptomic profiling is needed to determine whether adult Wilms tumours share the same pathogenesis as their paediatric counterparts (3, 23, 26, 27).

The newly investigated subject of the occurrence of Wilms tumour precursor lesions is also noteworthy. These are nephrogenic nests, which are simply the remaining components of cell nests after the embryogenic growth of renal tissue. Normally, the structures should disappear, but this does not always happen (1% of normal kidneys and 30–40% of kidneys with verified nephroblastoma, and with up to 100% proof in bilateral tumours). As a result, it is a developmental abnormality with a higher chance of developing nephroblastoma, particularly its bilateral versions. There is also a substantially higher probability of acquiring anaplasia (35% vs. 5% in “pure” nephroblastoma) (4, 28).

Because of the non-specificity of the findings and the absence of symptoms, most adult nephroblastomas are diagnosed in advanced stages with significant regional lymphadenopathy. The problem is also the rarity of this diagnosis, thus existing therapy options are “borrowed” from the juvenile equivalent (because randomised trials are not possible due to a limited number of probands). Therapy is governed by the National Wilms Tumour Study Group, according to which a double or triple combination of treatment modalities is preferred, i.e., primarily radical surgical resection, chemotherapy and (or) radiotherapy. However, the prognosis of adult Wilms tumours is generally considered to be extremely poor, owing to unfavourable histological findings and a lack of effective treatment guidelines (2, 3, 6, 10, 11, 15, 29–31).

## Conclusion

The main goal of this publication was to emphasise that, while it is generally a rare entity, similar challenging cases can occur in practise, and thus it is important to be aware of this type of tumour when making a differential diagnosis in cases with similar clinical and histopathological features.

## Data Availability

The original contributions presented in the study are included in the article/Supplementary material, further inquiries can be directed to the corresponding author.
