# Supplementary figures and images for: Blastemal predominant WT1 negative Wilms tumour of the young adult: a unique case report and review of the literature
Source: Front Med (Lausanne). 2025 Mar 19;12:1507011. doi: 10.3389/fmed.2025.1507011 (PMC11961890; doi:10.3389/fmed.2025.1507011)

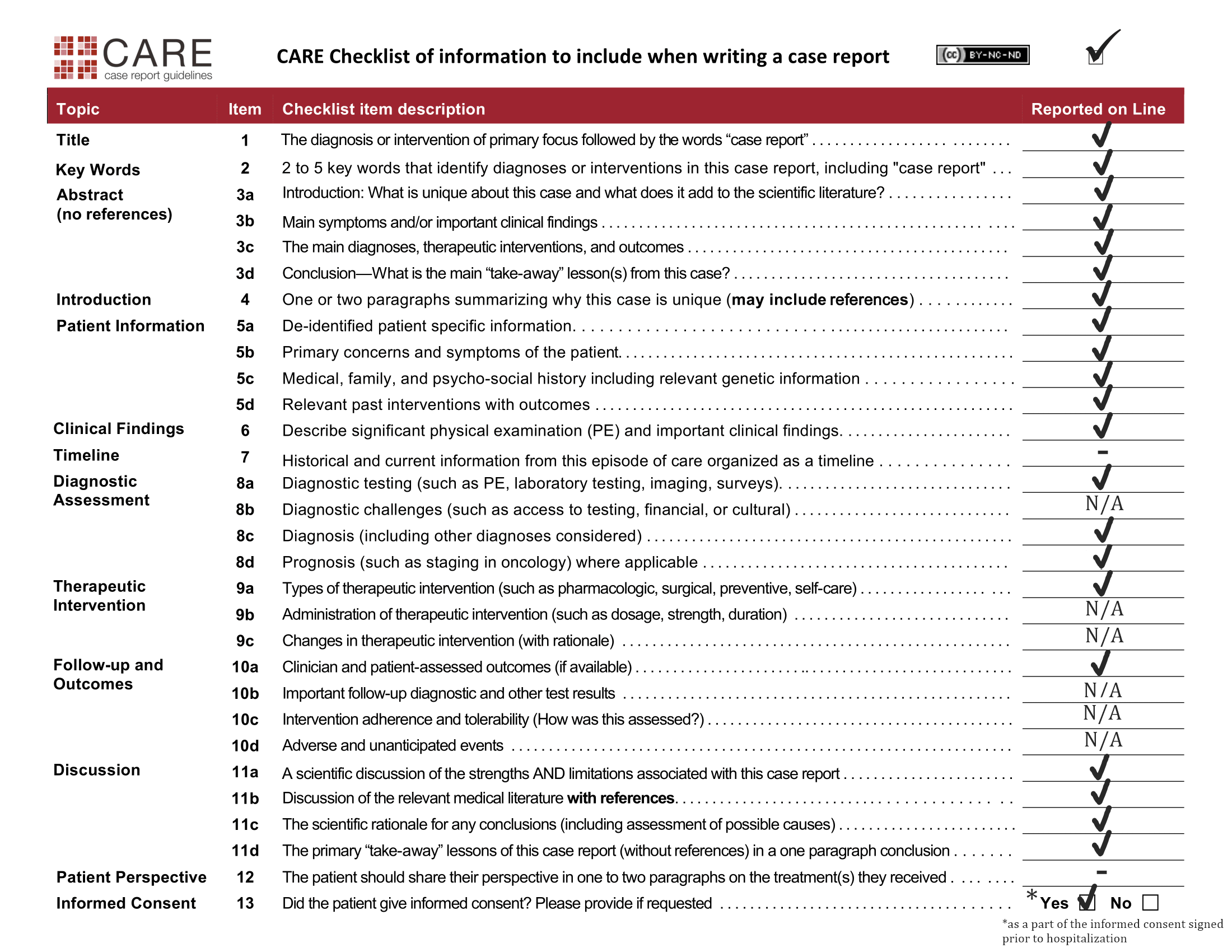

Supplement: Supplementary file 1 [file Image_1.png]
